# Down-regulation of CD81 in human cells producing HCV-E1/E2 retroVLPs

**DOI:** 10.1186/1753-6561-5-S8-P72

**Published:** 2011-11-22

**Authors:** Ana F Rodrigues, Miguel R Guerreiro, Rute Castro, Hélio A Tomás, Charlotte Dalba, David Klatzmann, Paula M Alves, Manuel J T Carrondo, Ana S Coroadinha

**Affiliations:** 1 Instituto de Biologia Experimental e Tecnológica/Instituto de Tecnologia Química e Biológica (IBET/ITQB-UNL), Apartado 12, P-2781-901 Oeiras, Portugal; 2 EPIXIS SA, 5 rue des Wallons, 75013 Paris, France; 3 AP-HP, Hôpital Pitié-Salpêtrière, Biotherapy, F-75013 Paris, France; 4 Faculdade de Ciências e Tecnologia/Universidade Nova de Lisboa (FCT/UNL), P-2825 Monte da Caparica, Portugal

## Background

Retroviral based biopharmaceuticals are used in numerous therapeutic applications, from gene therapy to vaccination [1-3]. Nonetheless, retroviruses are known to incorporate proteins from the host cell which may increase the vector immunotoxicity [4]. CD81, a membrane protein belonging to the tetraspanin superfamily has been recently identified as one of the host cell proteins majorly incorporated into Moloney Murine Leukemia Virus (MoMLV) derived vectors[5]. Therefore, CD81 was chosen as a target for studying the effects of host cell protein incorporation in the immunotoxic profile of retroviral vectors (RV). A human cell line, 293rVLP, producing retrovirus like particles (rVLPs) was used to prove the concept of customizing RV composition by manipulating cellular protein content, using the CD81 tetraspanin as a case study. rVLPs will be used as candidate vaccines for hepatitis C by displaying HCV-E1/E2 proteins.

## Materials and methods

Cell lines and culture media: 293rVLP is a HEK 293 (ATCC CRL-1573) based cell line established by stable transfection of pCeB plasmid [6] driving MoMLV gag-pol expression as described in [7]. 293T cells (ATCC CRL-11268) were used for producing and titer shRNA lentiviral vector stocks. All cells were maintained in Dulbecco’s modified Eagle’s medium (DMEM) (Gibco) 10% (v/v) Foetal Bovine Serum (FBS) (Gibco).

Plasmids and lentivirus production: Short hairpin (sh) RNA lentiviral vectors based on pLKO.1-puro [8] containing a puromycin resistance gene and targeting five different sequences on the human CD81 mRNA or a non-target (nt) shRNA vector were purchased from Sigma Mission® *RNAi* (Sigma). The lentiviral vector stocks were produced by transient co-transfection of 293T cells with pSPAX2, pMD2G (Addgene) and the respective pLKO.1-puro-shRNA.

Flow cytometry for CD81 detection: The presence of CD81 on the cell membrane was detected by flow cytometry using mouse monoclonal antibody against human CD81 and an Alexa FluorR 488 dye conjugated secondary antibody (Invitrogen).

Oxidative stress induction and cell viability: The susceptibility to oxidative stress injury was assessed by analyzing *tert*-butyl hydroperoxide induced death. Cell viability was quantified by the percentage of cells staining negative for PI (by flow cytometry).

RT activity: To determine MoMLV reverse transcriptase (RT) activity the RetroSys C-Type RT activity kit (Innovagen) was used.

Further details on Materials and Methods are in [9].

## Results and discussion

293rVLP cells producing rVLPs were used to evaluate the possibility of customizing vector composition by manipulating host protein content. CD81 tetraspanin was chosen to be down-regulated by shRNA, given that it is significantly incorporated into RV particles, conferring them a strong immunogenic character in mice. Each cell population, shCD81 8 to 12, as well as the non-target control, were analyzed for the presence of CD81 by flow cytometry (Table 1). CD81 down-regulation efficiency varied considerably among the five shRNA sequences, ranging from negligible in the case of shCD81 11 to a very strong silencing effect in shCD81 10 cell population, with more than 90% of cells being negative for CD81. ntshRNA population stained almost totally positive (94%) for CD81. To increase CD81 down-regulation, the shCD81 10 cell population was doubly infected with shCD81 9 and 12 (Table 1). Only 1.5% of the resulting cells were positive for CD81 which, considering the background values for the ntshRNA control (approx. 6%), suggested a totally CD81 silenced population.

**Table 1:** CD81 down-regulation in 293rVLP cells

		CD81^-^ cells (%)			
sh populations	ntshRNA	5.8			
	shCD81 8	20.4			
	shCD81 9	88.4			
	shCD81 10	92.8			
	shCD81 11	10.0			
	shCD81 12	31.0			
	shCD81 9+10+12	98.5			

		CD81^-^ cells (%)	Specific cell growth (h^-1^)	IC50 (µM *tert*-butyl hydroperoxide)	Specific RT productivity (10^-6^ ng/cell.h)

	293 rVLP	2.1	0.0201 ± 0.0002	3.3 ± 1.1	1.7 ± 0.3
	ntshRNA	0.8	0.020 ± 0.002	9.2 ± 1.4	2.3 ± 0.4
shCD81 9+10+12 clones	shCD81 #9	86.4	0.0124 ± 0.0004	15.4 ± 1.0	0.9 ± 0.2
	shCD81 #14	97.1	0.021 ± 0.002	4.5 ± 1.1	2.0 ± 0.4
	shCD81 #19	92.9	0.015 ± 0.001	6.5 ± 1.1	1.2 ± 0.2
	shCD81 #22	88.9	0.018 ± 0.001	6.0 ± 1.1	1.8 ± 0.4
	shCD81 #30	82.3	0.017 ± 0.001	5.6 ±1.0	1.4 ± 0.2

shCD81 9+10+12 cell population was cloned by limiting dilution and 30 clones were isolated. Four of those, #14, #19, #22 and #30 were chosen as representatives of the highest CD81 silencing (≥85%) and #9 as a representative of intermediate CD81 silencing. The rVLP production was analyzed by reverse transcriptase (RT) quantification in the supernatant; the cellular response to CD81 down-regulation and/or RNAi pathway activation was assessed by evaluating cell growth and cell death susceptibility under oxidative stress conditions (Table I). Cell population ntshRNA was used as negative control to distinguish CD81 silencing from RNAi pathway activation effects. The results were compared to the parental cell line, 293rVLP. With the exception of clone #14, all silenced clones presented lower cell growth. This effect, seemed to be attributed to CD81 down-regulation, as it was not detected in the ntshRNA control. Indeed, CD81 has been previously described to be implicated in proliferation and cytostasis of various cell types, including lymphocytes, fibroblasts, epithelial cells and astrocytes [10-13]. Lower half maximal inhibitory concentration values (IC_50_) under oxidative stress were observed for silenced cells, including the ntshRNA population, suggesting that the activation of RNAi pathway played a role in injury induced death susceptibility. From a bioprocessing point of view, increased susceptibility to stressful conditions, oxidative or other, should be a drawback. However, RNAi is faster and simpler than a knockout, and undeniably useful in preliminary stages. Additionally, for certain vital proteins, a total knock-out may not be possible.

To assess rVLP productivity in shCD81 clones the RT in the viral supernatant was quantified. Three of the five clones presented lower RT productivities, whereas clones #14 and #22 showed identical values to the non-target silenced control or the parental cell line. This suggested that CD81 down-regulation does not affect viral particles production. Finally, rVLP shCD81 #14, was used to produce rVLPs, which were concentrated, purified and analyzed for the presence of CD81 by Western blotting. The rVLPs were shown to be absent of CD81 demonstrating that it could be eliminated. These results suggest that, although naturally incorporated on RV, CD81 is not essential for the assembly or secretion of fully functional particles. Hepatitis C envelope proteins E1/E2 were successfully expressed in CD81 silenced cells and incorporated in rVLPs.

## Conclusions

In this work it was demonstrated that is possible to manipulate the composition of the host cell proteins incorporated into rVLPs. rVLps can be used as a epitope display platform and used in vaccine development therefore, CD81 was chosen since it is strongly immunogenic in mice. The results herein presented support that is possible to manipulate cellular protein expression, CD81 or others, as a tool to control RV composition and their immunogenic profile. Additionally, for gene therapy purposes, the removal immunotoxic proteins in the producer cell may improve the *in vivo* efficacy of retrovirus, lentivirus or other enveloped virus.
